# Supplementary material for: Emergency triage of brain computed tomography via anomaly detection with a deep generative model
Source: Nat Commun. 2022 Jul 22;13:4251. doi: 10.1038/s41467-022-31808-0 (PMC9307758; doi:10.1038/s41467-022-31808-0)
Supplement: Supplementary file 1 — Supplementary Information [file 41467_2022_31808_MOESM1_ESM.pdf]

Supplementary Materials

Supplementary Table 1. Baseline characteristics of the patients and image acquisition information according to the training, tuning, and validation sets

Supplementary Table 2. Emergency severity categories according to brain CT findings

Supplementary Table 3. Detection performance of CN-StyleGAN according to target severity

Supplementary Fig. 1: Confusion matrices of the classification of brain CTs based on emergency severity for assessing the performance of CN-StyleGAN

Supplementary Fig. 2: ROC curve analysis for assessing the performance of CN-StyleGAN for the detection of emergency cases by disease entity

Supplementary Fig. 3: False-positive and false-negative cases predicted by CN-StyleGAN

Supplementary Fig. 4: Training process of CN-StyleGAN

Supplementary Fig. 5: Inference method and anomaly scoring system for CN-StyleGAN

Supplementary Fig. 6: User interface for the clinical simulation test

Supplementary Table 1. **Baseline characteristics of the patients and image acquisition information according to the training, tuning, and validation sets**

**Characteristics**	**Training**	**Tuning**	**Internal validation**	**External validation**
Sample size	34,085	271	273	1,795
Age (year)	42.9 ± 19.6	58.1 ± 18.0	59.1 ± 17.6	60.3 ± 19.3
Sex				
Female	18,232 (53.5%)	143 (52.8%)	137 (50.2%)	875 (48.7%)
Male	15,853 (46.5%)	128 (47.2%)	136 (49.8%)	920 (51.3%)
Emergency severity				
Immediate		18 (6.6%)	18 (6.6%)	80 (4.5%)
Urgent		22 (8.1%)	23 (8.4%)	117 (6.5%)
Indeterminate		10 (3.7%)	10 (3.7%)	50 (2.8%)
Benign		59 (21.8%)	60 (22.0%)	436 (24.3%)
Normal		162 (59.8%)	162 (59.3%)	1,112 (61.9%)
Diseases in the emergency group		40	41	197
Brain mass-like lesion		10 (25%)	16 (39.0%)	20 (10.2%)
Acute infarction		6 (15%)	3 (7.3%)	39 (19.8%)
Hemorrhage		19 (47.5%)	18 (43.9%)	128 (65.0%)
Hydrocephalus		4 (10%)	2 (4.9%)	6 (3.0%)
Other diseases		1 (2.5%)	2 (4.9%)	4 (2.0%)
CT scanner	Siemens Healthcare (n = 19, 420)<br>· Definition<br>· SOMATOM Definition<br>· SOMATOM Definition Flash<br>· Definition AS<br>· SOMATOM Definition Edge<br>· SOMATOM Force<br>· SOMATOM Definition AS+<br>· SOMATOM Definition AS<br>· Sensation 16<br>GE Healthcare (n = 14,656)<br>· LifeSpeed Plus<br>· LightSpeed QX/i<br>· HiSpeed CT/i<br>· Optima CT660<br>· Discovery CT750 HD<br>· LightSpeed VCT<br>· LightSpeed16	Siemens Healthcare (n = 268)<br>· SOMATOM Definition Edge<br>· SOMATOM Definition Flash<br>· SOMATOM Definition AS+<br>GE Healthcare (n = 3)<br>· Discovery CT750 HD<br>· LightSpeed VCT	Siemens Healthcare (n = 270)<br>· SOMATOM Definition Edge<br>· SOMATOM Force<br>· SOMATOM Definition AS+<br>GE Healthcare (n = 3)<br>· Discovery CT750 HD<br>· LightSpeed VCT	Siemens Healthcare (n = 1751)<br>· SOMATOM Definition Edge<br>· SOMATOM Scope<br>GE Healthcare (n = 44)<br>· LightSpeed 16<br>· LightSpeed VCT

	Neurologica (n = 9)<br>· CereTom			
Slice thickness (mm)	· 4.8 (n = 14,316)<br>· 5 (n = 19,676)<br>· 10 (n = 93)	· 5 (n = 271)	· 5 (n = 273)	· 4.8 (n = 1,065)<br>· 5 (n = 730)

Data are presented as the mean ± standard deviation or number of cases (%). Abbreviations: CT, computed tomography

Supplementary Table 2. Emergency severity categories according to brain CT findings

Categories		Definition
Emergency	Immediate	CT findings suggest a critical, life-threatening condition that requires immediate medical or surgical treatment.
		· Brain tumor with a mass effect resulting in midline shift and herniation<br>· Intracranial hemorrhage with a mass effect resulting in midline shift and herniation<br>· Extensive subarachnoid hemorrhage<br>· Hypoxic encephalopathy<br>· Large territorial or malignant acute infarction
	Urgent	CT findings suggest a current non-life-threatening condition that requires rapid treatment to prevent deterioration.
		· Intracranial hemorrhage without a mass effect<br>· Focal acute infarction<br>· Tumor without a mass effect, such as midline shift and herniation<br>· Marked hydrocephalus (Evans' index > 0.4)<br>· Unruptured giant aneurysm
Non-emergency	Indeterminate	CT findings suggest that prompt treatment is not required but further workup or follow-up is required.
		· Indeterminate small hypodense cerebral lesions<br>· Incidental pituitary adenoma, small meningioma, or suspected small aneurysm<br>· Hydrocephalus (0.4 ≥ Evans' index > 0.34)
	Benign	CT findings suggest that no further workup is required in the emergency department.
		· Severe brain atrophy, arachnoid cyst, encephalomalacia, leukoaraiosis, or postoperative change
	Normal	Normal

Abbreviation: CT, computed tomography

Supplementary Table 3. **Detection performance of CN-StyleGAN according to target severity.**

The results are presented as the area under the receiver operating characteristic (ROC) curve (AUC), sensitivity, specificity, and accuracy with 95% confidence intervals. The threshold was derived using the maximum value of Youden's index for the ROC curve using the tuning dataset.

**Target group**	**Performance of CN-StyleGAN**			
**Internal validation**	**AUC**	**Sensitivity**	**Specificity**	**Accuracy**
Normal brain CT	0.91 (0.88–0.94)	0.70 (0.63–0.77)	0.94 (0.92–0.97)	0.85 (0.82–0.88)
Urgent brain CT	0.77 (0.71–0.83)	0.52 (0.36–0.69)	0.78 (0.74–0.82)	0.75 (0.71–0.79)
Immediate brain CT	0.96 (0.94–0.99)	0.94 (0.88–1.00)	0.78 (0.74–0.82)	0.79 (0.75–0.83)
Emergency brain CT (urgent and immediate)	0.85 (0.81–0.89)	0.71 (0.60–0.82)	0.78 (0.74–0.82)	0.77 (0.73–0.80)
**External validation**				
Normal brain CT	0.88 (0.87–0.90)	0.56 (0.54–0.59)	0.94 (0.93–0.95)	0.80 (0.78–0.81)
Urgent brain CT	0.81 (0.79–0.84)	0.68 (0.61–0.75)	0.81 (0.80–0.83)	0.80 (0.79–0.82)
Immediate brain CT	0.95 (0.93–0.96)	0.93 (0.90–0.99)	0.81 (0.80–0.83)	0.81 (0.80–0.82)
Emergency brain CT (urgent and immediate)	0.87 (0.85–0.89)	0.78 (0.74–0.82)	0.81 (0.80–0.83)	0.81 (0.80–0.82)

Abbreviations: AUC, area under the receiver operating characteristic curve; CT, computed tomography

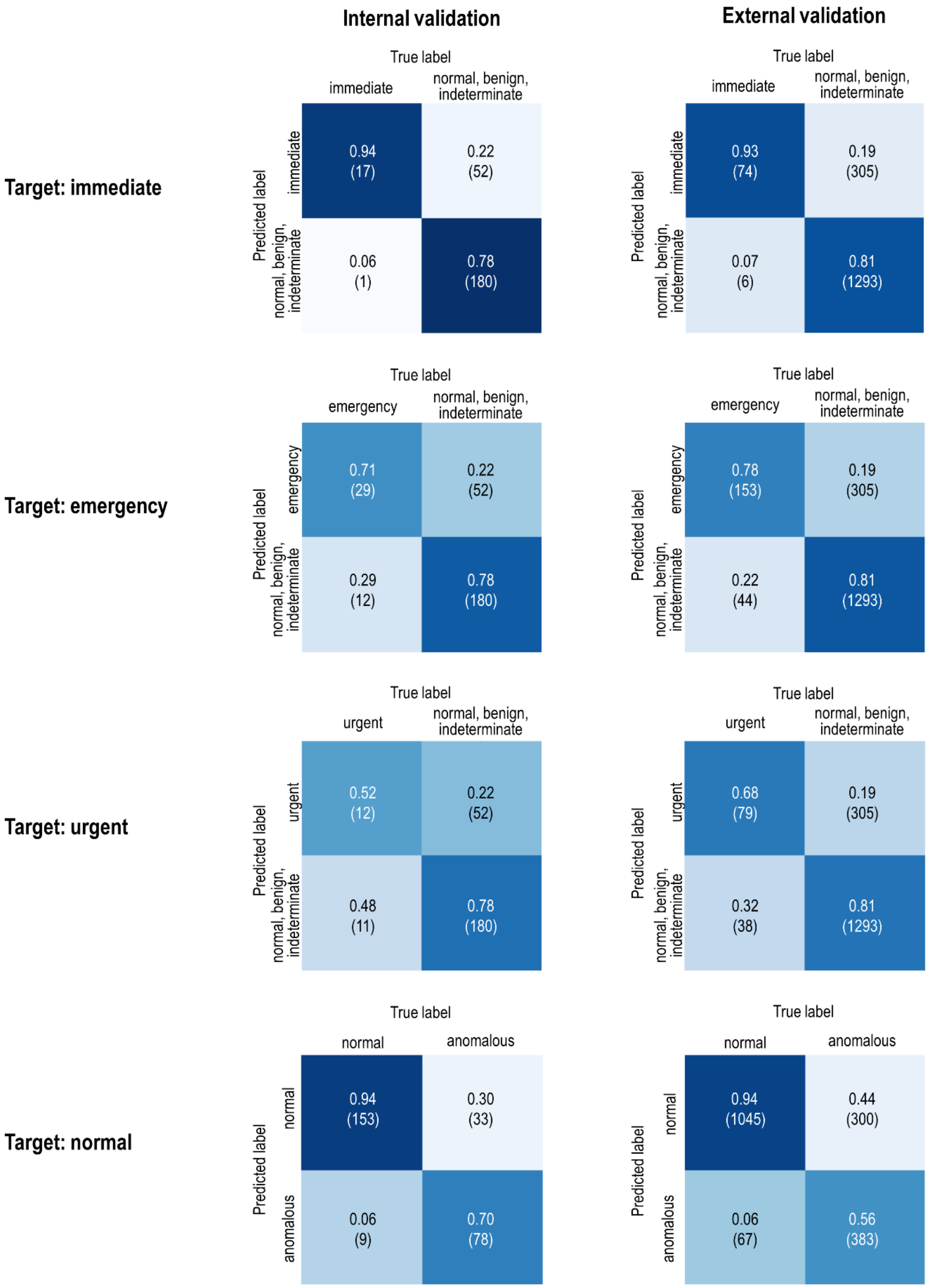

Supplementary Fig. 1: Confusion matrices of the classification of brain CTs based on emergency severity for assessing the performance of CN-StyleGAN. Confusion matrices showing the number of brain CT images for the reference and predicted diagnosis of CN-StyleGAN according to the

emergency severity of brain CT images in the internal and external validation datasets. In each confusion matrix, the target groups include immediate, emergency, urgent, and normal cases. Emergency cases are defined as cases requiring immediate or urgent intervention, regardless of the neurological entity. Anomalous cases are defined as a complementary set of normal cases, including benign, indeterminate, urgent, and immediate cases.

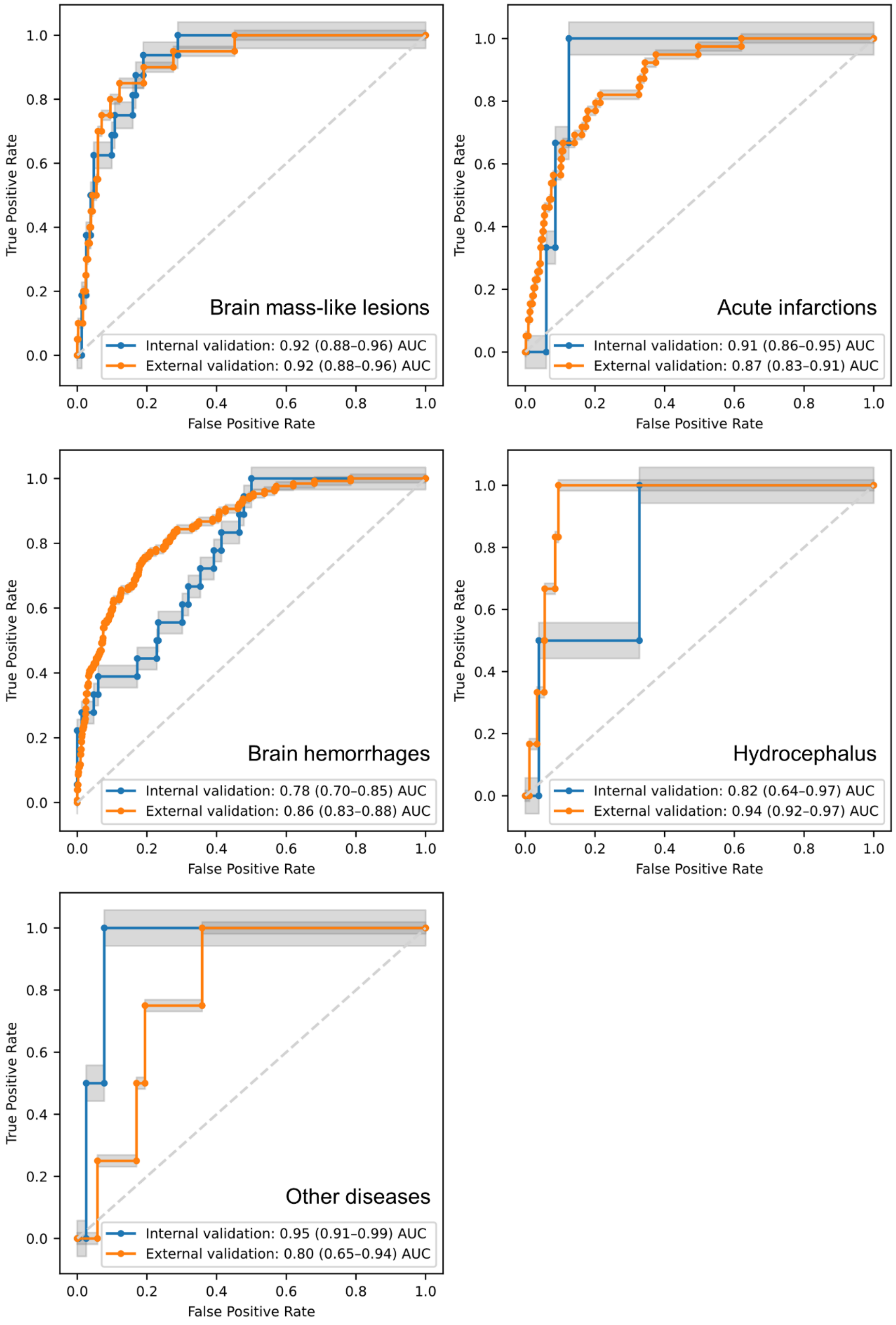

Supplementary Fig. 2: ROC curve analysis for assessing the performance of CN-StyleGAN for the detection of emergency cases by disease entity. Data are presented as mean AUC values with 95% CI.

a

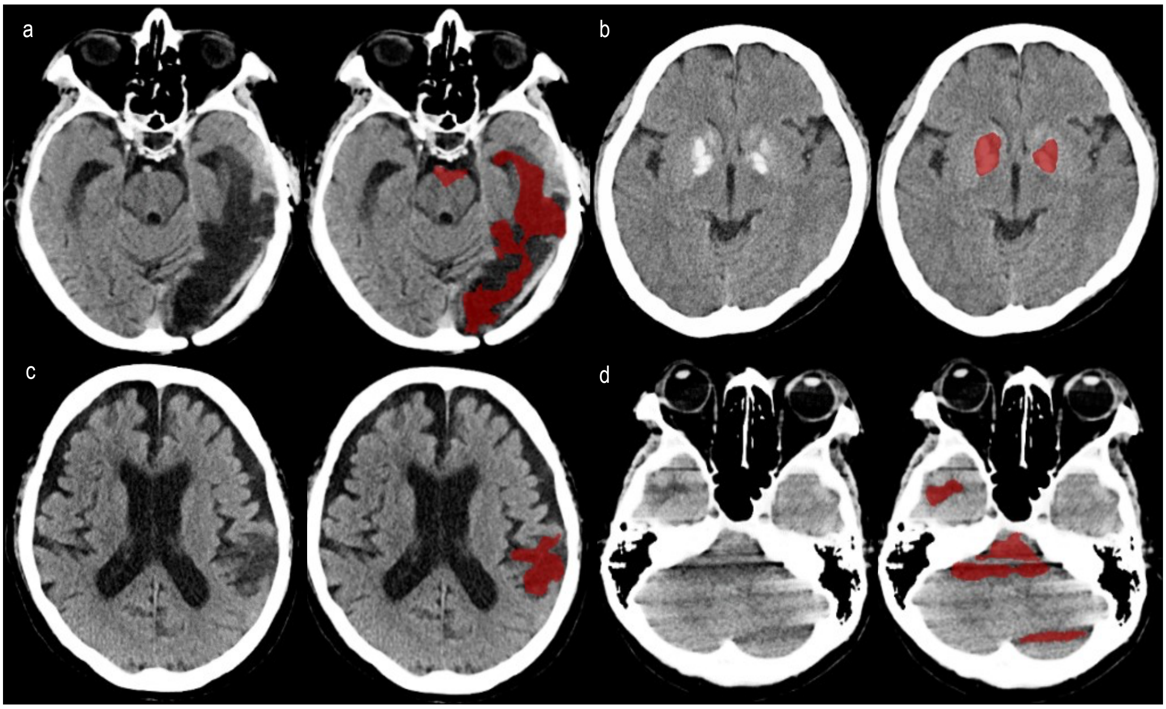

b

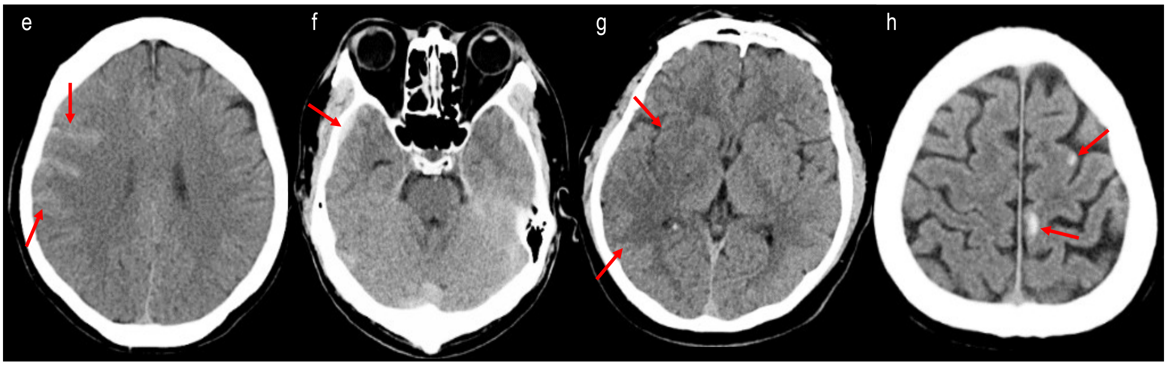

Supplementary Fig. 3: False-positive and false-negative cases predicted by CN-StyleGAN

a The false-positive cases include anomalous cases that do not require urgent or immediate treatment. In each example, the left-sided image represents the original input image, and the right-sided image represents the image with predicted abnormal regions. **b** Most false-negative cases consisted of brain lesions with a relatively small volume or a subtle attenuation change (arrows). a, encephalomalacia (old infarction); b, intracranial calcification not related to normal aging; c, normal age-related prominent sulci; d, motion artifact; e, traumatic subarachnoid hemorrhage; f, subdural hemorrhage; g, early-stage acute infarction; and h, small, calcified metastases.

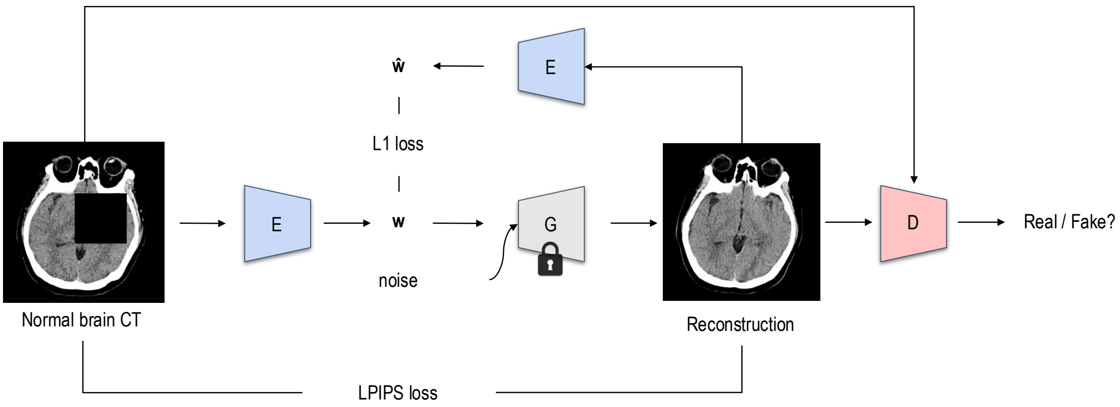

Supplementary Fig. 4: Training process of CN-StyleGAN

The architecture of CN-StyleGAN includes a style-based generator (**G)**, discriminator (**D)**, and style-based encoder neural network (**E**). The training dataset containing normal brain CT axial slices was used to jointly train **D** and **E,** while the pre-trained weights of **G** were kept constant. The model learned to reconstruct a query brain CT image as the closest-normal brain CT image. Note that the input brain CT image was randomly erased for the model to learn the context of normal brain CT images by filling in the missing region.

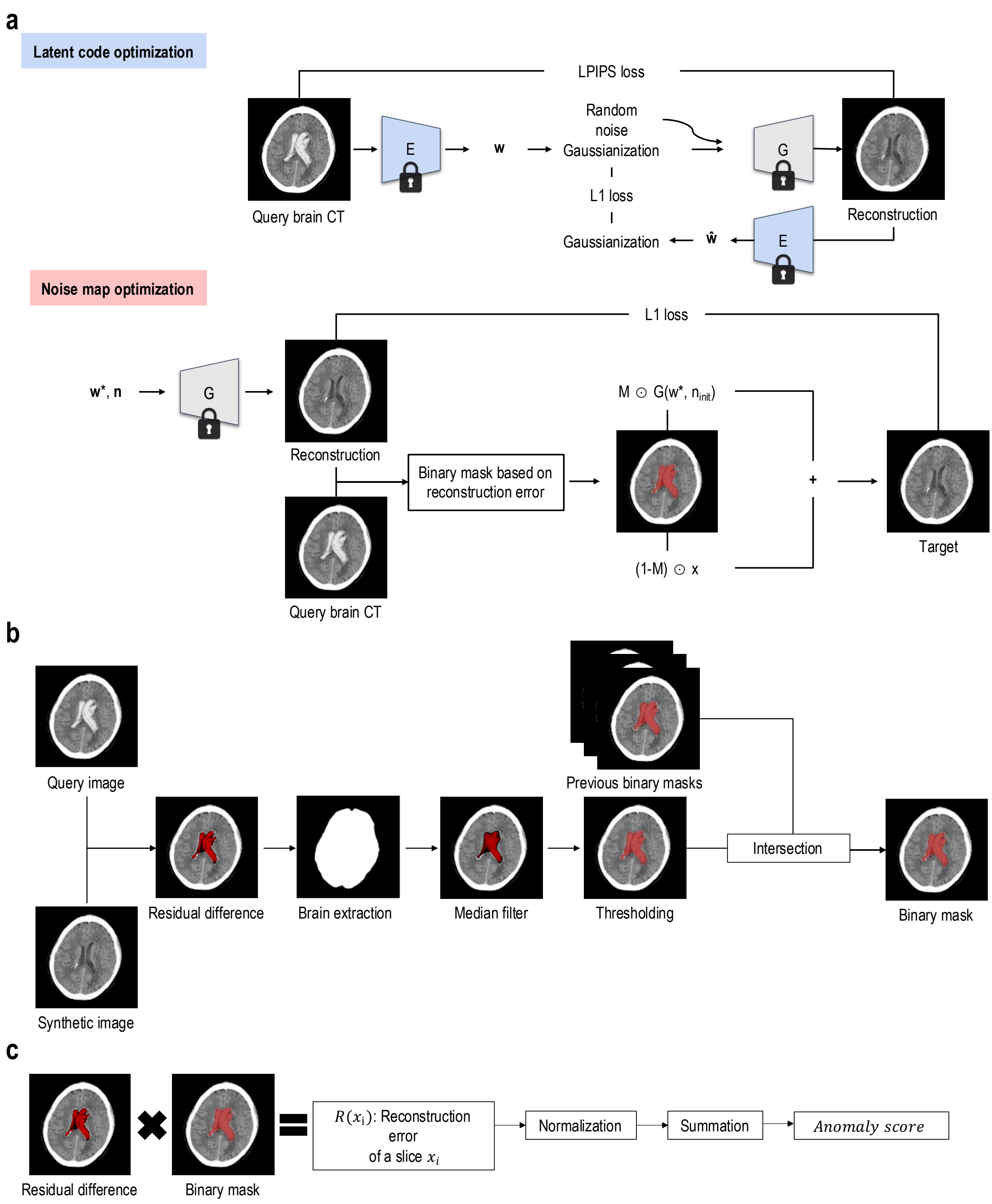

Supplementary Fig. 5: Inference method and anomaly score system of CN-StyleGAN

This figure demonstrates the inference method and anomaly scoring system of CN-StyleGAN. **a,** The inference method of CN-StyleGAN. Given a brain CT slice, $\boldsymbol{x}$, the latent vector, $\boldsymbol{w}$, was initialized as $E(\boldsymbol{x})$, and the noise maps, $\boldsymbol{n}$, were initialized from the unit normal distribution. After the latent vector optimization, the noise maps were optimized. Masked noise optimization was proposed for the noise

map optimization. **b,** Derivation of the binary mask in the masked noise optimization process. The residual difference between an input image and the reconstructed image was brain-extracted, median-filtered, and thresholded. Moreover, the false positives in the binary mask were reduced because of the intersections between the binary masks in the previous optimization steps. **c**, The calculation process of the anomaly scoring system. The reconstruction error of a slice was derived as the binary masked density error between the input slice and the reconstructed slice. This reconstruction error was normalized, slice by slice, based on the slice order, using the reconstruction error statistics (mean and SD of normal brain CT images) from the training dataset. Finally, this normalized per-slice reconstruction error of 32 slices for a CT scan was summed to determine the anomaly score.

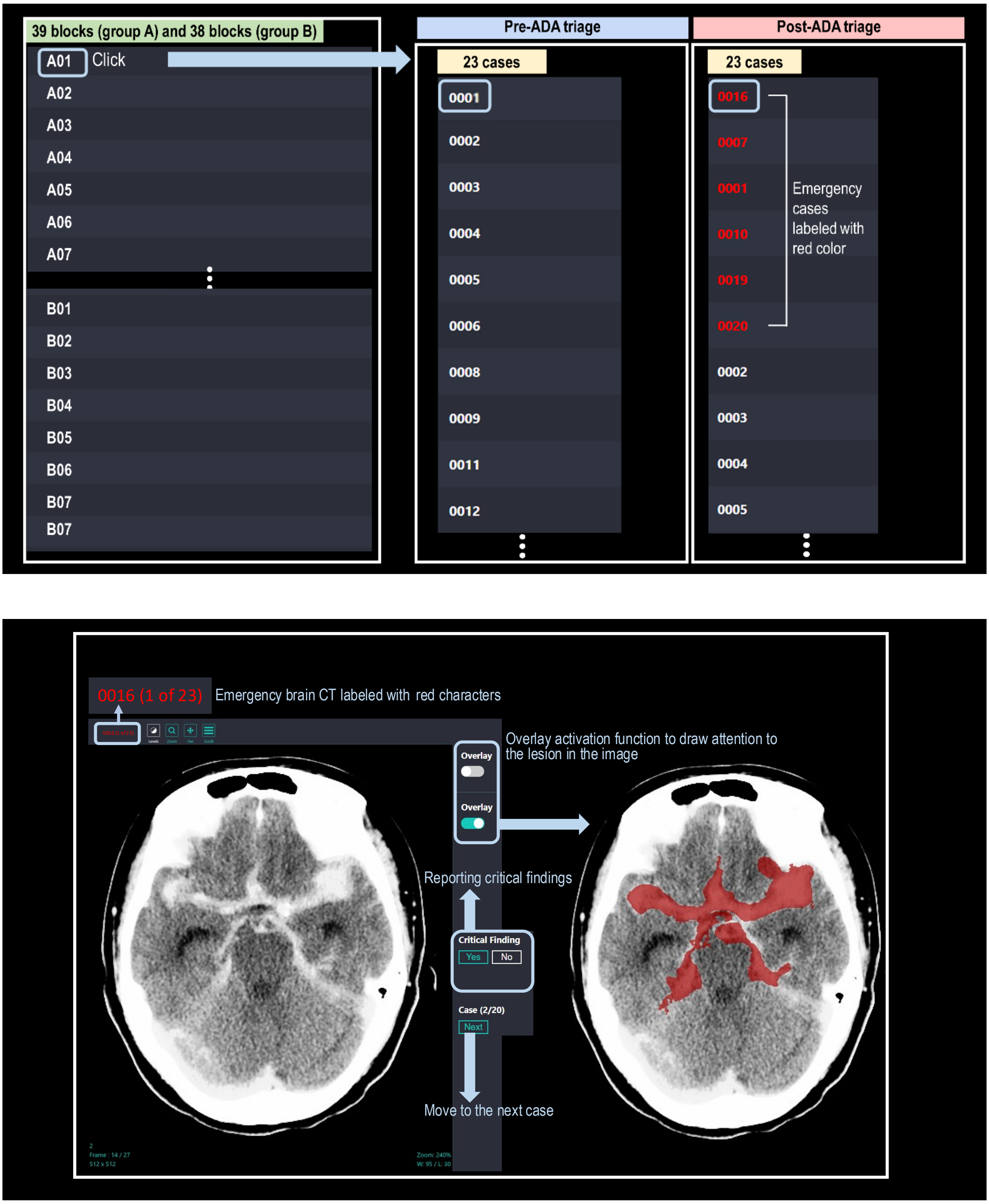

Supplementary Fig. 6: **User interface for the clinical simulation test**

The web-based user interface shown here provides the radiology worklists of brain CT images and displays Digital Imaging and Communications in Medicine (DICOM) images. In the first screen, the readers can select a block in a top-to-bottom order. In the next screen, the worklists of brain CT images in the selected block are observed. The readers can open each brain CT image according to the assigned

order. The opening times of each block and each brain CT image are automatically recorded. The readers can adjust the window level of the images and can zoom in to magnify the images. After the readers determine the presence or absence of emergency CT findings, they click the "Critical Finding" button to report the CT findings. If the readers click the button "Next," the user interface will automatically move to the next case. The time is automatically recorded upon clicking the buttons. Pre-ADA triage, the user interface provides worklists of randomly ordered brain CT images. Post-ADA triage, the ADA reprioritizes brain CT images in the worklists and labels emergency cases with red color. The user interface provides the overlay activation function. The readers can see the lesion attention (mask overlay) predicted by the ADA by clicking the button "Overlay."
