# Supplementary material for: Emergency triage of brain computed tomography via anomaly detection with a deep generative model
Source: Nat Commun. 2022 Jul 22;13:4251. doi: 10.1038/s41467-022-31808-0 (PMC9307758; doi:10.1038/s41467-022-31808-0)
Supplement: Supplementary file 2 — Reporting Summary [file 41467_2022_31808_MOESM2_ESM.pdf]

nature portfolio

Corresponding author(s): Gil-Sun Hong, Namkug Kim

Manuscript number: NCOMMS-21-32482C

Last updated by author(s): Jun 28, 2022

Reporting Summary

Nature Portfolio wishes to improve the reproducibility of the work that we publish. This form provides structure for consistency and transparency in reporting. For further information on Nature Portfolio policies, see our Editorial Policies and the Editorial Policy Checklist.

Statistics

For all statistical analyses, confirm that the following items are present in the figure legend, table legend, main text, or Methods section.

n/a	Confirmed	
☐	☒	The exact sample size (*n*) for each experimental group/condition, given as a discrete number and unit of measurement
☐	☒	A statement on whether measurements were taken from distinct samples or whether the same sample was measured repeatedly
☐	☒	The statistical test(s) used AND whether they are one- or two-sided<br>*Only common tests should be described solely by name; describe more complex techniques in the Methods section.*
☒	☐	A description of all covariates tested
☒	☐	A description of any assumptions or corrections, such as tests of normality and adjustment for multiple comparisons
☐	☒	A full description of the statistical parameters including central tendency (e.g. means) or other basic estimates (e.g. regression coefficient) AND variation (e.g. standard deviation) or associated estimates of uncertainty (e.g. confidence intervals)
☐	☒	For null hypothesis testing, the test statistic (e.g. F, t, r) with confidence intervals, effect sizes, degrees of freedom and P value noted<br>*Give P values as exact values whenever suitable.*
☒	☐	For Bayesian analysis, information on the choice of priors and Markov chain Monte Carlo settings
☐	☒	For hierarchical and complex designs, identification of the appropriate level for tests and full reporting of outcomes
☒	☐	Estimates of effect sizes (e.g. Cohen's d, Pearson's r), indicating how they were calculated

Our web collection on statistics for biologists contains articles on many of the points above.

Software and code

Policy information about availability of computer code

Data collection	The NLP algorithm (pyConTextNLP version 0.6.0.5) was utilized for labeling for healthy brain CT scans was used in this study.
Data analysis	Analyses were performed using Python version 3.8.5 (sklearn 0.23.2; Python Software Foundation), R version 4.1.0 (R Foundation for Statistical Computing), and ggplot2 version 3.6.3. All source codes are available at https://github.com/seungjunlee96/emergency-triage-of-brain-computed-tomography-via-anomaly-detection-with-a-deep-generative-model.

For manuscripts utilizing custom algorithms or software that are central to the research but not yet described in published literature, software must be made available to editors and reviewers. We strongly encourage code deposition in a community repository (e.g. GitHub). See the Nature Portfolio guidelines for submitting code & software for further information.

Data

Policy information about availability of data

All manuscripts must include a data availability statement. This statement should provide the following information, where applicable:

- Accession codes, unique identifiers, or web links for publicly available datasets
- A description of any restrictions on data availability
- For clinical datasets or third party data, please ensure that the statement adheres to our policy

Sample images or derived data supporting the findings of this study are available with the corresponding author on reasonable request. The raw experimental and clinical data are provided as Source Data. The brain CT images for the development and validation of the model are not publicly available because they contain private patient health information. For reasonable purposes including reproducing results in this study, researchers can request the corresponding authors, Gil-Sun Hong and Namkug Kim, with approval of the Institutional Ethics Committee of Asan Medical Center. The requests will be processed in 30 business days.

Field-specific reporting

Please select the one below that is the best fit for your research. If you are not sure, read the appropriate sections before making your selection.

☒ Life sciences ☐ Behavioural & social sciences ☐ Ecological, evolutionary & environmental sciences

For a reference copy of the document with all sections, see nature.com/documents/nr-reporting-summary-flat.pdf

Life sciences study design

All studies must disclose on these points even when the disclosure is negative.

Sample size	We developed the deep learning model using brain CT images of healthy patients and validate the performance of the model in emergency department patients with suspicious neurological diseases. Prior to the study, the effect size was not known. Therefore, we aimed to include approximately as many patients in this study.
Data exclusions	For the training dataset, only normal CT scans were included. Therefore, we excluded CT scans of 79,060 postoperative CT scans, 78,713 abnormal CT scans, and 5,180 CT scans that were not available for automatic download using the in-house system.<br><br>For the validation tests, CT images of consecutive patients from the emergency department of internal and external institutions were collected. 6 CT scans from the internal dataset were excluded due to download error.
Replication	Results were replicated using internal and external emergency screening cohorts, which consisted of consecutively collected scans at the emergency department.
Randomization	The tuning and test datasets were randomized using built-in random module in Python.
Blinding	When investigating the effect of the model as a radiologic triage system (the clinical simulation test), all readers were blinded to all clinical information.

Reporting for specific materials, systems and methods

We require information from authors about some types of materials, experimental systems and methods used in many studies. Here, indicate whether each material, system or method listed is relevant to your study. If you are not sure if a list item applies to your research, read the appropriate section before selecting a response.

Materials & experimental systems

n/a	Involved in the study	
☒	☐	Antibodies
☒	☐	Eukaryotic cell lines
☒	☐	Palaeontology and archaeology
☒	☐	Animals and other organisms
☐	☒	Human research participants
☒	☐	Clinical data
☒	☐	Dual use research of concern

Methods

n/a	Involved in the study	
☒	☐	ChIP-seq
☒	☐	Flow cytometry
☒	☐	MRI-based neuroimaging

Human research participants

Policy information about studies involving human research participants

Population characteristics	Demographics related to the training data (n = 34,085) are follows:<br>- Healthy patients, mean age ± standard deviation [SD], 42.9 ± 19.6 years; 18,232 females [53.5 %].<br><br>Demographics related to the tuning dataset (n = 271) are as follows:<br>- emergency screening cohorts<br>- Mean age ± SD, 58.1 ± 18.0 years, 143 females [52.8%]<br>- Emergency brain CT scans (14.8%, n = 40)<br>- Diseases: brain mass-like lesion (25%, n = 10), acute infarctions (15%, n = 6), intracranial hemorrhages (47.5%, n = 19), hydrocephalus (10%, n = 4), and other diseases (2.5%, n = 1)<br><br>Demographics related to the internal validation dataset (n = 273) are as follows:<br>- emergency screening cohorts<br>- Mean age ± SD, 59.1 ± 17.6 years, 137 females [50.2%]<br>- Emergency brain CT scans (11.0%, n = 197)<br>- Diseases: brain mass-like lesion (39.0%, n = 16), acute infarctions (7.3%, n = 3), intracranial hemorrhages (43.9%, n = 18), hydrocephalus (4.9%, n = 2), and other diseases (4.9%, n = 2)<br><br>Demographics related to the external validation data (n = 1,795) are as follows:<br>- Consecutive emergency screening cohorts<br>- Mean age ± SD, 60.3 ± 19.3 years, 875 females [48.7%]<br>- Emergency brain CT scans (11.1%, n = 195)<br>- Diseases: brain mass-like lesion (10.2%, n = 20), acute infarctions (19.8%, n = 39), intracranial hemorrhages (65.0%, n = 128), hydrocephalus (3%, n = 6), and other diseases (2%, n = 4)
Recruitment	For the model development, a total of 197,038 non-contrast brain CT scans and paired radiology reports were retrospectively collected from patients who visited an urban, tertiary, academic hospital (Asan Medical Center) between January 1, 2000, and August 31, 2018. After five iterations of the data curation process, the final training dataset comprised 34,085 normal brain CT scans from healthy patients. Briefly, the NLP-based data curation process consisted of three steps: 1) keyword selection for CT abnormal findings, 2) excluding brain CT scans based on these keywords using a natural language processing (NLP) algorithm, 3) Analysis of the results of the data curation process. For the tuning and internal validation test, after six cases were excluded due to download errors, 544 non-contrast brain CT scans of ED patients were consecutively collected from Asan Medical Center in February 2019. For the external validation test, 1,795 non-contrast brain CT scans from ED patients were consecutively collected from Gangneung Asan Hospital from January 1, 2019, to May 31, 2019. Internal and external validation datasets represent real-world data that were consecutively collected from ED patients with neurologic symptoms and acquired from diverse CT machines and scanning protocols.
Ethics oversight	This retrospective study was conducted in accordance with the principles of the Declaration of Helsinki and current scientific guidelines. The Institutional Review Boards (IRBs) of Asan Medical Center (2019-0795) and Gangneung Asan Hospital (GNAH 2020-01-006) approved the study protocol. They waived the requirement for informed patient consent, given the minimal risk to subjects in the retrospective imaging study and the impracticality of obtaining informed consent from large numbers of patients retrospectively.

Note that full information on the approval of the study protocol must also be provided in the manuscript.
